# Midshaft Clavicle Fractures Treated Nonoperatively Using Figure-of-Eight Bandage: Are Fracture Type, Shortening, and Displacement Radiographic Predictors of Failure?

**DOI:** 10.3390/diagnostics10100788

**Published:** 2020-10-05

**Authors:** Jacopo Tagliapietra, Elisa Belluzzi, Carlo Biz, Andrea Angelini, Ilaria Fantoni, Manuela Scioni, Mario Bolzan, Antonio Berizzi, Pietro Ruggieri

**Affiliations:** 1 Orthopaedic Clinic, Department of Surgery, Oncology and Gastroenterology DiSCOG, University of Padova, 35128 Padova, Italy; tagliapietra.j@gmail.com (J.T.); elisa.belluzzi@gmail.com (E.B.); andrea.angelini@unipd.it (A.A.); ilaria.fantoni89@gmail.com (I.F.); antonio.berizzi@unipd.it (A.B.); pietro.ruggieri@unipd.it (P.R.); 2 Musculoskeletal Pathology and Oncology Laboratory, Department of Surgery, Oncology and Gastroenterology DiSCOG, University of Padova, 35128 Padova, Italy; 3 Department of Statistical Sciences, University of Padova, 35121 Padova, Italy; scioni@stat.unipd.it (M.S.); mario.bolzan@unipd.it (M.B.)

**Keywords:** midshaft clavicle fractures, figure-of-eight bandage, shortening and displacement

## Abstract

As there are no clear and unique radiographic predictors of healing disturbances for acute midshaft clavicle fractures, their treatment is still controversial. The aim of the study was to evaluate in midshaft clavicle fractures treated nonoperatively if fracture type (FT), shortening, and displacement, assessed before and after figure-of-eight bandage (F8-B) application, could be considered prognostic factors of delayed union and nonunion. One hundred twenty-two adult patients presenting a closed displaced midshaft clavicle fracture, managed nonoperatively with an F8-B, were enrolled. FT, initial shortening (IS), and initial displacement (ID) were radiographically evaluated at diagnosis, and both residual shortening (RS) and displacement (RD) were measured after F8-B application. The patients were followed up 1, 3, 6, and 12 months post-injury. Multivariate statistical analysis was performed. RD should be considered as radiological predictor of sequelae. Further, an RD equal to 104% of clavicle width was identified as an optimal cut-off point to distinguish between healed and unhealed fractures, and 140% between delayed union and nonunion. Our data pointed out the effectiveness of the F8-B in reducing fracture fragments and restoring clavicular length. In midshaft clavicle fractures of adults, fracture comminution and clavicular shortening did not influence bone healing. On the contrary, RD has been shown as the most likely predictor of both delayed union and nonunion.

## 1. Introduction

Clavicle fractures are common, ranging from 2.6 to 4% of all fractures and from 35 to 44% of fractures of the shoulder girdle [1,2,3]. Young adults and middle-aged patients are generally more affected, with sport injuries and recreational activities representing the most described traumatic patterns [1,4]. Up to 80% of clavicle fractures occur at the midshaft [5], due to its narrow cross-section and the absence of ligament or muscle attachment [6,7,8]. Around 15% of clavicle fractures occur at the lateral third, and 5% on the medial third [6,7,8,9]. Most midshaft clavicle fractures are displaced, as a result of the muscular action and the weight of the arm [6,8]. Treatment of displaced midshaft clavicle fractures is still controversial [10,11,12,13,14,15]. Traditionally, these fractures have been treated nonoperatively, even in cases of severe displacement or comminution, based on the high capacity of healing and remodeling of the clavicle [16,17,18]. However, recent studies have shown high rates of nonunion and suboptimal clinical outcomes for displaced clavicle fractures managed nonoperatively [7,19,20,21]. Since Novak et al. suggested surgical treatment in the presence of severe displacement with no body contact in 2004, an increased number of clavicle fractures have been treated surgically, particularly in cases of substantial displacement, shortening, or comminution [22]. On the other hand, a recent systematic review by Jørgensen et al. affirmed that clavicular shortening and comminution are doubtful predictors of nonunion, while displacement was the only reliable predictor. Thus, the authors undersized the role of surgery for clavicle fractures, suggesting that it should be restricted to severely displaced lesions and assuming that an extensive use of surgery would lead to overtreatment [23]. Indeed, despite several randomized trials and recent meta-analysis, there is currently no consensus on surgical indications. Open fractures, skin tenting with the potential for progression to open fracture, “floating shoulder”, and associated neurovascular injuries represent the only accepted indications for surgery in acute lesions. There are still controversies in cases of comminution, severe displacement, and shortening; in the latter cases, in fact, the choice of treatment seems to be strongly influenced by the professional background and experience of the orthopedic surgeon [10,14,24,25,26,27,28].

The aim of this study was to define reliable radiographic predictors of delayed union and nonunion in adult midshaft clavicle fractures treated with a figure-of-eight bandage (F8-B). Our hypothesis was that fracture comminution, displacement, and shortening could be possible radiographic predictors of healing disturbances, particularly when assessed after the F8-B application. Moreover, the effectiveness of the F8-B technique in improving fracture shortening and displacement was evaluated in the present study.

## 2. Materials and Methods

### 2.1. Study Population

Adult patients (18–65 years) admitted to the Emergency Department (ED) of our Institution between January 2012 and December 2015 with a midshaft clavicle fracture (Association for Osteosynthesis/Orthopedic Trauma Association (AO/OTA) 15.2 [29]) were retrospectively recruited for this study. At our level-I healthcare trauma centre, initial treatment of midshaft clavicle fractures is highly standardized. Open fractures and/or displaced fractures with skin tenting are treated surgically by open reduction and internal fixation (ORIF). Further, we advocate surgery in cases of floating shoulder, polytrauma, concomitant cervical spine or thoracic trauma, and neurovascular injuries. In all other cases, a nonoperative approach is preferred, either an arm-supporting sling or an F8-B. The first is employed for undisplaced fractures and low-demanding patients, otherwise the F8-B is applied.

In this study were included active adult patients presenting to our ED with a closed displaced midshaft clavicle fracture managed nonoperatively with an F8-B who respected and completed the entire follow-up program (as shown below). Patients with simultaneous fractures of the shoulder girdle or history of previous ipsilateral upper arm major trauma, oncologic patients receiving chemotherapy and/or radiotherapy, patients receiving immunosuppressive treatment, patients with rheumatic diseases, diabetes mellitus, or vascular diseases, patients with pathologic fractures, and low-demanding patients were excluded. This study was approved by the Local Institutional Ethics Committee (Prot. Num. 0058228; Tit. II Cl.10 fasc.141) and was performed in accordance with the ethical standards of the 1964 Declaration of Helsinki as revised in 2000 and those of Good Clinical Practice.

### 2.2. Patient Assessment and Evaluation of the Radiographic Outcomes

**Figure 1:**
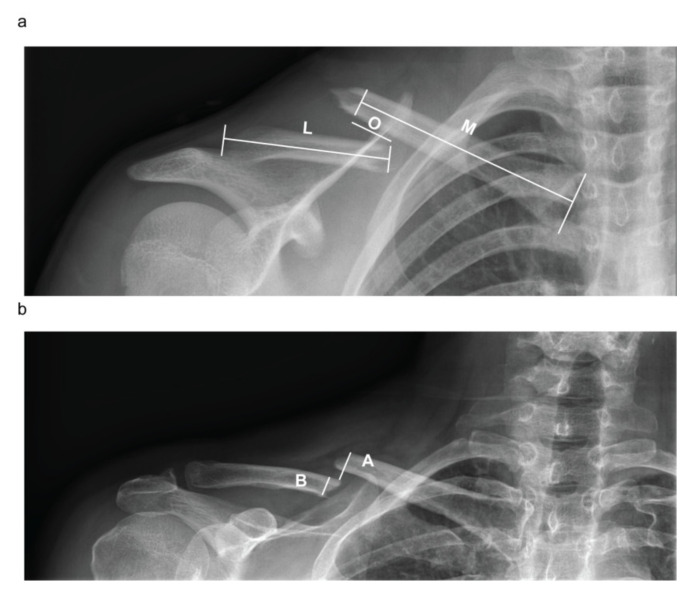
Clavicle shortening and displacement evaluation. (**a**) Clavicular shortening, defined as the overlap of proximal and distal fragments, was assessed as a percentage of the ipsilateral clavicular length on a standard antero-posterior view $=\frac{O}{M+L}\%$. (**b**) The amount of fragment displacement, defined as the amount of vertical translation, was assessed as a percentage of the clavicle shaft width at the fracture site on a 20° cephalic tilt view of the clavicle. $D=\frac{A}{B}\%$ O = overlapping; M = medial clavicle fragment length; L = lateral clavicle fragment length; A = cortex-to-cortex distance; B = clavicle width; S = shortening; D = displacement.

For each patient, fracture type (FT), initial shortening (IS), and initial displacement (ID) were radiographically evaluated at diagnosis at the ED of our institution. Sequentially, both residual shortening (RS) and displacement (RD) were measured immediately after the application of the F8-B. The FT was established according to the AO/OTA Classification [29]. Clavicular shortening, defined as the overlap of proximal and distal fragments, was assessed as a percentage of the ipsilateral clavicular length on a standard antero-posterior view (Figure 1a). The amount of fragment displacement, defined as the amount of vertical translation, was assessed as a percentage of the clavicle shaft width at the fracture site on a 20° cephalic tilt view of the clavicle (Figure 1b).

### 2.3. Nonoperative Treatment and Follow-Up Program

The bandage was applied after the initial radiographic evaluation of the fracture and maintained for four to six weeks, depending on the presence of clinical and radiographic signs of healing. Patients were adequately informed about how to address their daily life activities with the bandage. Moreover, they were instructed on how to maintain correct position of the bandage, how to retension it, and how to avoid complications, such as axillary pressure ulcers or compression of the neurovascular bundle. To avoid skin irritation due to the bandage in the axillary area, patients were allowed to add some form of padding. Each patient had a clinical and radiographic evaluation at 7 and 15 days from the first visit to assess the bandage position, possible changes of fracture fragments, or any other clinical problems during the clinical activities of our unit. Where a significant worsening of fragment displacement leading to skin tenting occurred, surgical treatment was proposed. In cases of poor tolerance to the F8-B, an arm-supporting sling was applied. In all other cases, treatment with the F8-B was continued. Radiographic follow-up was assessed until the fracture union, while clinical follow-up was performed at 1, 3, 6, and 12 months post-injury. Delayed union was defined as the absence of clinical and radiographic signs of fracture consolidation within three months, and nonunion as the absence of fracture consolidation within six months [19,20,30]. Where diagnosis of nonunion was unclear, a CT scan was performed to ensure the absence of bony healing at the fracture site.

### 2.4. Statistical Analysis

For continuous variables, the median and the range were provided. For categorical variables, frequency and percentage distribution were performed. The Kruskal–Wallis test was applied in order to compare quantitative variables between the groups. If the Kruskal–Wallis test was significant, a post hoc analysis was performed through the Dunn test with adjustment for *p* value by Bonferroni method, and a Chi-square (χ^2^) test was performed to compare categorical variables. Multinomial logistic regression was applied to model the multivariate relationship between a categorical variable and some predictors. Results of the multinomial logistic regression analysis were expressed in the form of z-coefficients and relative *p*-value. For significant variables, also the relative risk ratio, that is, the risk relative to the base category, was considered. To evaluate the general goodness of fit of the multinomial model, a McFadden’s pseudo R-squared was considered. A *p* < 0.05 was considered significant. A ROC curve analysis according to the Youden criterion was performed to determine the optimal cut-off point for binary classifier. All the analyses were performed with R [31].

## 3. Results

### Patient Characteristics

**Figure 2:**
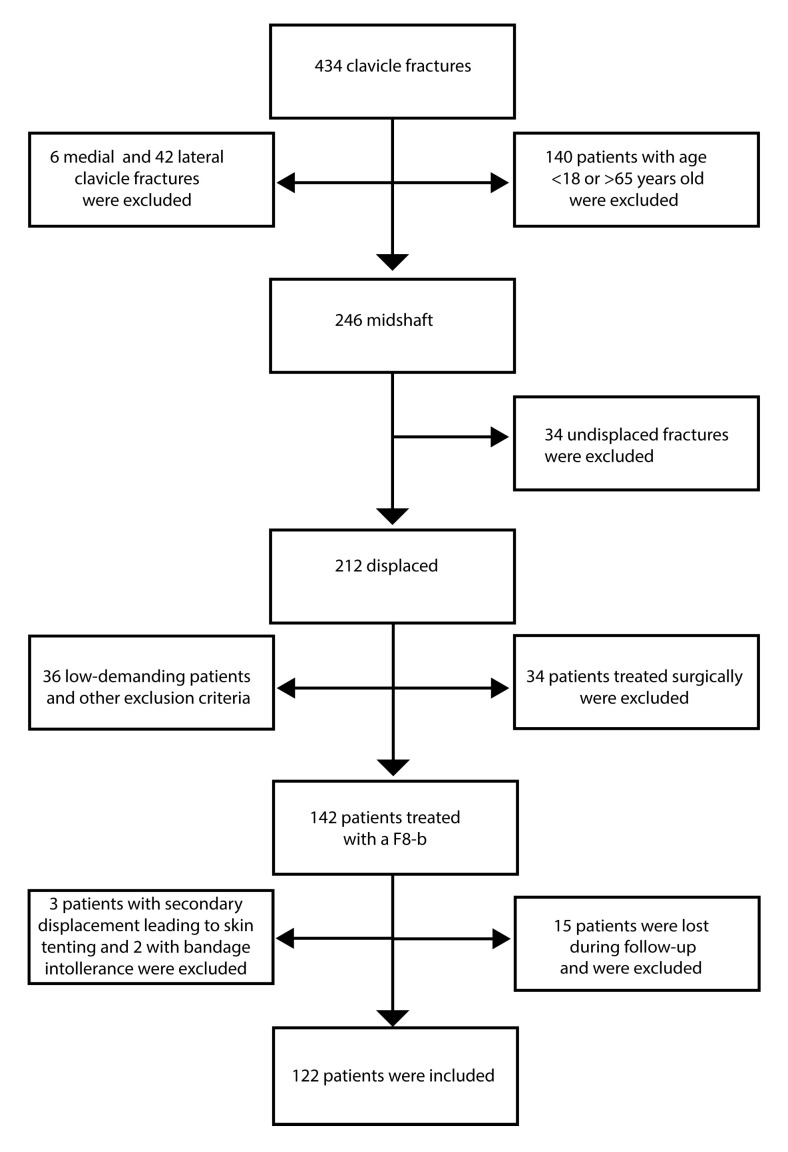
Flowchart illustrating patient selection.

A total of 142 patients with a displaced midshaft clavicle fracture were treated with an F8-B. Three patients were then excluded as they experienced secondary displacement leading to skin tenting, requiring surgical treatment. Two patents refused to continue the treatment as they did not tolerate the bandage and were excluded. Fifteen patients were excluded as they were lost during the follow-up. A final cohort of 122 patients who met the inclusion criteria was enrolled in the study (Figure 2). Baseline characteristics of the patients are depicted in Table 1. Eighty-one patients (66.4%) showed fracture healing within 12 weeks, 24 (19.7%) patients between 12 and 24 weeks (delayed union), and 17 (13.9%) presented nonunion. Fifty-two fractures (42.6%) were classified as type A, while 70 (57.4%) as type B fractures.

Median IS and RS were both 0% (range 0–12), while median ID and RD were 114.5% (range 16–230) and 100% (range 10–185), respectively (Table 1).

On the basis of the clinical and radiologic evaluation, patients were divided into three groups: healed fractures, delayed union, and nonunion. No differences were found regarding the age of the patients belonging to the three groups (*p* = 0.720) (Table 2). No significant differences were found also with respect to the FT (*p* = 0.10).

**Figure 3:**
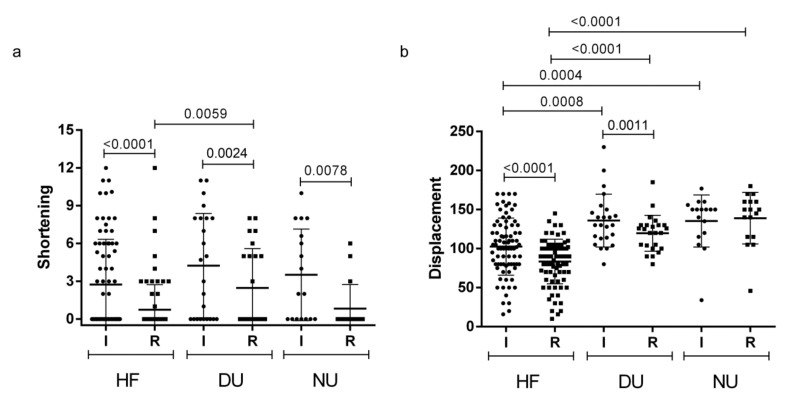
Initial and residual shortening (**a**) and displacement (**b**). HF = healed fractures, DU = delayed union, NU = nonunion, I = initial, R = residual.

No differences were found regarding IS comparing the three groups (*p* = 0.15), while a difference was found for RS (*p* = 0.014), specifically between healed fractures and delayed union (*p* = 0.0059) (Figure 3a). Interestingly, a decrease of the amount of clavicle shortening was found comparing IS and RS for each group (*p* < 0.0001, *p* = 0.024, *p* = 0.0078, respectively) (Figure 3a).

A difference was found comparing ID between the three groups (*p* < 0.0001), in particular between healed fractures and delayed union (*p* = 0.0008) and between healed fractures and nonunion (*p* = 0.0004) (Table 2). A difference was also found comparing RD between the groups (*p* < 0.0001), particularly between healed fractures and delayed union (*p* < 0.0001), and between healed fractures and nonunion (*p* < 0.0001). A decrease of the amount of fragment displacement was found comparing ID and RD in both the healed and the delayed fracture group (*p* < 0.0001 and *p* = 0.011, respectively) (Figure 3b).

Multinomial logistic regression was applied to identify which factors were associated with a major risk of treatment failure. The reference category was assumed to be healed fractures. The McFadden’s pseudo R-squared equals 0.389, demonstrating a good model fit [32]. Considering delayed union with respect to healed fractures, assumed to be the reference category, only RD was proven to be a significant determinant. The relative risk ratio (RRR) equals 1.060 (95% CI = 1.023, 1.098). This means that if RD increases by one unit, it corresponds to a 6% increase of the relative risk of getting a delayed union instead of a healed fracture, given the other variables in the model are held constant. Considering nonunion, always with respect to healed fractures, RD (RRR = 1.122 95% CI = 1.065, 1.182) and FT (RRR = 0.174 95% CI = 0.033, 0.929) were found to be significant predictors. As before, we can state that if RD increases by one unit, it corresponds to a 12.2% increase of the relative risk of a nonunion instead of a healed fracture. Regarding the FT, type B fractures had an 82.6% reduction in relative risk of nonunion compared to those with a type A fracture.

**Figure 4:**
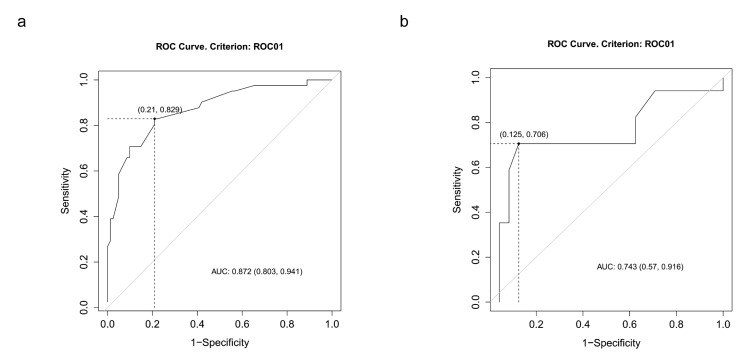
ROC curve. (**a**) Receiver operating characteristic (ROC) curve for residual displacement for the diagnosis of nonhealing fractures. (**b**) Receiver operating characteristic (ROC) curve for residual displacement for the discrimination between nonunion and delayed union.

A ROC curve analysis was performed to identify cut-off points for RD to discriminate the treatment failure. A first ROC curve (Figure 4a) was plotted to discriminate between healed fractures and the unhealed ones (delayed union and nonunion). An RD equal to 104% was identified as an optimal cut-off point to discriminate between the two groups, using Youden’s index maximization as the criterion or, equivalently, the misclassification rate minimization. The area under the curve (AUC) for the proposed test equals 0.872, which indicates a good accuracy of the test. A second ROC curve (Figure 4b) was plotted to discriminate between delayed union and nonunion, using the same criterion. An RD of 140% was identified as an optimal cut-off point to distinguish between delayed union and nonunion. The AUC equals 0.743, indicating a fair accuracy of the test.

## 4. Discussion

The purpose of this study was to verify if FT, shortening, and displacement could be considered reliable radiographic predictors of delayed union and nonunion in displaced midshaft clavicle fractures. No differences were found between the three groups regarding age and FT. In particular, incidence of delayed union or nonunion did not diverge significantly among different AO/OTA fracture types, showing that comminution was not predictive of healing disturbances, in accordance with Jørgensen et al. [23].

The main limitation of our retrospective study is that fractures were assessed using standard radiographs, which do not allow the evaluation of the accurate tridimensional fragment position. A limitation is the low number of patients, particularly the small sample size of the nonunion group. Another limitation is the lack of a control group. However, our study is in line with other published studies in terms of sample size as well as of the incidence of nonunion [3,33,34,35].

Regarding shortening and displacement, the literature mostly evaluated them in terms of absolute values or compared them to the contralateral clavicle [3,36,37,38]. However, since intersubject and intrasubject variability in terms of clavicle width and length has been reported [14,39,40], in the present work relative shortening and displacement were considered. In fact, we decided to define the fragment displacement as a percentage on the ipsilateral midshaft clavicle width, while shortening was normalized as a percentage on the ipsilateral clavicle length. Using these relative measures, we took into account the aforementioned anatomical variability of the clavicle. In addition, using this method, standard X-rays of the injured clavicle are sufficient to evaluate fracture pattern, without needing contralateral clavicle views or making use of more refined, but expensive, radiological exams.

No differences were found comparing the IS between the groups, but a significance was found comparing RS between healed and delayed fractures.

Clavicle shortening has not been shown to be a predictive risk factor either of delayed union or of nonunion in our cohort of patients, supporting the data published by Jørgensen et al. [23]. However, it is an important feature to be considered, as according to many authors, it seems to be related to worse functional outcome [6,41,42,43,44].

Regarding clavicle displacement, a difference was found between the three groups comparing both the ID and RD. Multilogistic analysis revealed that RD after the application of the F8-B was significantly related to an increased risk of healing disturbances, thus representing the most likely predictor of delayed union and nonunion. In the literature, only ID has been related to the incidence of healing disturbances [22,23,35,38]. On the contrary, our study pointed out that the attention should be moved to RD, which should be considered as the most likely radiological predictive factor of sequelae. To the best of our knowledge, this is the first study evaluating RD after bandage application as a potential predictor of healing disturbances.

Indeed, we pointed out that the rate of delayed union and nonunion increases proportionally with the amount of RD, reporting two cut-off points. An RD greater than 104% significantly increases the risk of delayed union, while an RD greater than 140% is associated with a higher rate of nonunion.

Recently, the effectiveness of the F8-B has been questioned; it reportedly does not give superior results compared to a sling in terms of clinical and radiographic outcomes [45,46], and causes greater discomfort, especially during the first days [47]. Randomized controlled trials comparing the use of the two bandages are needed. However, our data stress the effectiveness of the F8-B in reducing fracture fragments and restoring clavicular length. In analyzing clavicle shortening, a significant decrease comparing IS and RS in each group was found and a consistent decrease was also noticed between ID and RD in the healed and delayed union groups, supporting the effectiveness of the bandage. In particular, the F8-B should be maintained in patients showing an immediate radiographic improvement after bandage application, as they have the highest potential of successful healing. On the other hand, when a severe displacement, particularly if higher than 140%, persists after bandage application, the presence of any mechanical factors which impair fragment reduction should be suspected, for instance, the interposition of soft tissues. In these cases, the use of the bandage is ineffectual and other treatments should be considered.

Further studies need to be performed on a wide cohort of patients to confirm our findings. In addition, the absence of a consensus in how to assess both shortening and displacement using the standard clavicle X-ray series interferes with the comparison between different studies.

## 5. Conclusions

In conclusion, in contrast to the initial hypothesis of the study, in adult midshaft clavicle fractures treated with an F8-B, fracture comminution and clavicular shortening did not influence bone healing. On the contrary, residual fragment displacement has been shown as the most likely predictor of both delayed union and nonunion.

## Figures and Tables

**Table 1:** Overall patient characteristics.

	Patients Enrolled (122)
Age, mean (SD) Median (range)	37 (12.7) 38 (62–18)
Sex, Female (%) Male (%)	19 (15.6) 103 (84.4)
Type of fractures (FT), number (%) A1 A2 A3 B1 B2 B3	9 (7.4) 31 (25.4) 12 (9.8) 10 (8.2) 23 (18.9) 37 (30.3)
Type of fractures (FT), number (%) A B	52 (42.6) 70 (57.4)
Fracture healed within 12 weeks, number (%) Delayed union Nonunion	81 (66.4) 24 (19.7) 17 (13.9)
Initial shortening % (IS), median (range)	0 (12–0)
Residual shortening % (RS), median (range)	0 (12–0)
Initial displacement % (ID), median (range)	114.5 (230–16)
Residual displacement % (RD), median (range)	100 (185–10)

**Table 2:** Nonunion rate by type of fracture, amount of displacement and shortening.

Variable	Healed Fracture (81/122)	Delayed Union (24/122)	Nonunion (17/122)	*p*-Value (Kruskal–Wallis)
Age, mean (SD) Median (range)	37.4 (11.7) 38 (61–18)	36.2 (14.5) 36.5 (62–18)	39.5 (14.5) 38 (60–18)	0.720
Type of fracture, % (number/total *n* =122) A1 A2 A3 B1 B2 B3	66.7 (6/9) 67.7 (21/31) 25 (3/12) 60 (6/10) 86.7 (20/23) 67.6 (25/37)	11.1 (1/9) 19.4 (6/31) 33.3 (4/12) 30 (3/10) 8.7 (2/23) 21.6 (8/37)	22.2 (2/9) 12.9 (4/31) 41.7 (5/12) 10 (1/10) 4.3 (1/23) 10.8 (4/37)	0.494
Type of fracture (FT), % (number/total *n* =122) A B	57.7 (30/52) 72.8 (51/70)	21.15 (11/52) 18.6 (13/70)	21.15 (11/52) 8.6 (6/70)	χ^2^ 0.10
Initial shortening % (IS), median (range)	0 (12–0)	3.85 (11–0)	2 (10–0)	0.15
Residual shortening % (RS), median (range)	0 (12–0)	0 (8–0)	0 (6–0)	0.014 * 0.0059 § 1 # 0.06571
Initial displacement % (ID), median (range)	102 (170–16)	130 (230–80)	150 (177–34)	<0.0001 * 0.0008 § 0.0004 # 0.890
Residual displacement % (RD), median (range)	90 (145–10)	122.5 (185–80)	150 (180–46)	<0.0001 * <0.0000 § <0.0000 # 0.2425

* = healed fractures vs. delayed, § = healed fractures vs. nonunion, # = delayed vs. nonunion.
